# The Antibacterial Effects of Supermolecular Nano-Carriers by Combination of Silver and Photodynamic Therapy

**DOI:** 10.3389/fchem.2021.666408

**Published:** 2021-04-15

**Authors:** Gui-ning Feng, Xiao-tao Huang, Xin-lin Jiang, Ting-wei Deng, Qiu-xia Li, Jie-xia Li, Qian-ni Wu, Song-pei Li, Xian-qiang Sun, Yu-gang Huang, Ai-ping Qin, Lu Liang, Ji-jun Fu

**Affiliations:** ^1^The Key Laboratory of Molecular Target & Clinical Pharmacology, School of Pharmaceutical Sciences, The First Affiliated Hospital of Guangzhou Medical University and the Fifth Affiliated Hospital of Guangzhou Medical University, Guangzhou Medical University, Guangzhou, China; ^2^School of Pharmaceutical Sciences, Shenzhen University, Shenzhen, China

**Keywords:** bacteria, antimicrobial photodynamic therapy, silver, supermolecular nano-carrier, synergistic efficacy

## Abstract

The over-use of antibiotics has promoted multidrug resistance and decreased the efficacy of antibiotic therapy. Thus, it is still in great need to develop efficient treatment strategies to combat the bacteria infection. The antimicrobial photodynamic therapy (aPDT) and silver nanoparticles have been emerged as effective antibacterial methods. However, the silver therapy may induce serious damages to human cells at high concentrations and, the bare silver nanoparticles may rapidly aggregate, which would reduce the antibacterial efficacy. The encapsulation of sliver by nano-carrier is a promising way to avoid its aggregation and facilitates the co-delivery of drugs for combination therapy, which does not require high concentration of sliver to exert antibacterial efficacy. This work constructed a self-assembled supermolecular nano-carrier consisting of the photosensitizers (PSs), the anti-inflammatory agent and silver. The synthesized supermolecular nano-carrier produced reactive oxygen species (ROS) under the exposure of 620-nm laser. It exhibited satisfying biocompatibility in L02 cells. And, this nano-carrier showed excellent antibacterial efficacy in *Escherichia coli* (*E. coli*) and *Staphylococcus aureus* (*S. aureus*) as indicated by bacterial growth and colony formation. Its antibacterial performance is further validated by the bacteria morphology through the scanning electron microscope (SEM), showing severely damaged structures of bacteria. To summary, the supermolecular nano-carrier TCPP-MTX-Ag-NP combining the therapeutic effects of ROS and silver may serve as a novel strategy of treatment for bacterial infection.

## Introduction

For treating the bacteria infection, antibiotics are the most widely used agents. The clinical application of antibiotics has made an obvious impact on the treatment of infectious disease and has sharply reduced mortality. However, the over-use of antibiotics has promoted multidrug resistance and decreased the efficacy of antibiotic therapy (Andersson and Hughes, 2010; Baym et al., 2016). Thus, it is still in great need to develop efficient strategies to combat the bacteria infection.

Recently, photodynamic therapy (PDT) that utilizes photosensitizers (PSs) and light irradiation to generate cytotoxic reactive oxygen species (ROS) has been supposed to be a promising option in bacteria ablation (de Melo et al., 2013; Hamblin, 2016; Galstyan et al., 2017; Wainwright et al., 2017; Deng et al., 2019; Qiu et al., 2019; Zhang et al., 2020). Antimicrobial PDT (aPDT), also named as photodynamic inactivation (PDI), uses visible light or near-infrared (NIR) light of appropriate wavelength to convert oxygen to cytotoxic ROS. ROS, such as superoxide anions, singlet oxygen and hydroxyl radicals, are highly reactive with biological molecules including lipids, proteins and nucleic acid and, cause irreversible oxidative damage of bacterial cells. As ROS can target numerous molecules, the microbes are very unlikely to develop resistance.

The metallic nanoparticles, especially the silver (Ag) nanoparticles have been demonstrated to be effective antibacterial agents with broad antibacterial activity (Agarwal et al., 2010; Mahmoudi and Serpooshan, 2012; Neibert et al., 2012; Durmus and Webster, 2013; Mei et al., 2013; Shah et al., 2013; Agnihotri et al., 2015; Borrelli et al., 2015; Lim et al., 2015; Kyaw et al., 2017; Ximing et al., 2017; Kim et al., 2018; Liu et al., 2018, 2019; Gasmalla et al., 2019; Zhang et al., 2019, 2020; He et al., 2020; Horue et al., 2020; Tong et al., 2020). Silver nanoparticles can increase the permeability of bacterial membrane and cell wall and, penetrate into the cytoplasm. Silver nanoparticles were reported to inactive the signaling cascades required for bacterial survival and colony formation (Tang and Zheng, 2018), such as respiratory enzymes responsible for DNA and RNA replication and, lead to the death of bacteria (Morones-Ramirez et al., 2005). In addition, the Ag^+^ released from silver nanoparticles is supposed to be cytotoxic to bacteria by interacting with cell membrane and enzymes (Xiu et al., 2012; Vilela et al., 2017; Xie et al., 2017). However, both silver nanoparticles and Ag^+^ exhibited serious damages to mammalian cells at high concentrations (Miller et al., 2015; Richter et al., 2015). Moreover, the bare silver nanoparticles tend to aggregate, which will decrease the antibacterial efficacy (Tong et al., 2019). In this work, the hybrid materials with good dispersion property were constructed to address these issues.

This work aims to combine aPDT and silver into hybrid nano-carrier to combat bacteria. In addition, the methotrexate (MTX) is also included in the nano-carrier to treat the inflammatory reaction associated with bacterial infection (Lebugle et al., 2002; Alam et al., 2017; Yang et al., 2017; Duan and Li, 2018; Trujillo-Nolasco et al., 2019; Sun et al., 2020). To address the aforementioned issues of bare silver nanoparticles, different nano-carriers have been constructed, such as metal-organic frameworks (MOFs) (Ximing et al., 2017; Zhang et al., 2019, 2020), hybrid metallic nanoparticles (Mahmoudi and Serpooshan, 2012; Durmus and Webster, 2013; Mei et al., 2013; Shah et al., 2013; Agnihotri et al., 2015; Lim et al., 2015; Kyaw et al., 2017; Kim et al., 2018; Liu et al., 2018; Gasmalla et al., 2019; He et al., 2020; Tong et al., 2020) and mesoporous nanoparticles (Liu et al., 2019). However, the above nano-carriers have some drawbacks, for instance, the low drug loading capacity (<10%) (Cai et al., 2015) and the complexity of designing a multicomponent nano-carrier (containing at least the carrier and two or more compounds). The drug loading capacity could be maximized if the drug itself is one of the compositions of nano-carrier. Instead of using any carrier materials, the self-assembly of drug molecules into nanoparticles is a straightforward method to prepare nanomedicines. Compared to traditional carrier-based drug delivery systems, these carrier-free nanodrugs could not only avoid the possible toxicity of the carrier materials but also possess the advantages of simplicity of preparation and high drug payload (Zhou et al., 2013; Li et al., 2015, 2017, 2020; Hou et al., 2018; Xue et al., 2020; Yuan et al., 2020).

To treat bacterial infection through the antibacterial effects of aPDT and silver in combination with the anti-inflammatory effects of MTX, this work constructed a self-assembled supermolecular nano-carrier consisting of the photosensitizer 4,4′,4″,4‴-(Porphine-5,10,15,20-tetrayl) tetrakis (benzoic acid) (TCPP), MTX, gallic acid (GA) and Poloxamer 407 (Pluronic F127). These four components self-assembled into nanostructure TCPP-MTX-NP and, the nano-carrier was further modified by silver element through the reduction reaction by GA to construct the Ag-doped TCPP-MTX-Ag-NP. The detailed structure of the supermolecular nano-carrier was analyzed and the ROS generation property was validated. The antibacterial effect of the synthesized nanocarrier against the Gram-positive *S. aureus* and the Gram-negative *E. coli* were evaluated.

## Materials and Methods

### Materials

The reagents methotrexate (MTX), 4,4′,4″,4‴-(Porphine-5,10,15,20-tetrayl) tetrakis (benzoic acid) (TCPP), gallic acid (GA), silver nitrate (AgNO_3_), sodium dodecyl sulfate (SDS), 1,3-Diphenylisobenzofuran (DPBF), paraformaldehyde (4%) solution and 3-(4,5-dimethylthiazol-2-yl)-2,5-diphenyltetrazolium bromide (MTT) were purchased from Aladdin Company (Shanghai, China). The acetonitrile (chromatographic grade), N,N-Dimethylformamide (DMF), *d*_6_- Dimethyl sulfoxide (*d*_6_-DMSO), chloroform-d (CDCl_3_), Trifluoroacetic acid-d (CF_3_COOD) and Deuterium oxide (D_2_O) were purchased from Macklin Company (Shanghai, China). The Calcein/PI kit was bought from Beyotime Corporation (Shanghai, China). The Poloxamer 407 (Pluronic F127) was kindly gifted by Basf Corporation (Shanghai, China).

### Preparation of the Supermolecular Nano-Carriers

Four milligram of MTX and 4.0 mg of TCPP were dissolved in 300 μL of DMF. One milligram of AgNO3 was dissolved in 100 μL of deionized water. 4.5 mg of GA and 15.0 mg of F127 were dissolved in 4 mL of deionized water under stirring. Then, the above mixture of MTX and TCPP was quickly added into the solution of GA and F127 under stirring. The solution changed from colorless to dark brown. Then, the AgNO3 solution was added. The mixture was stirred for another 8 h before being sent to dialysis (cutting molecular weight: 10 kDa) in deionized water or saline for 24 h to get the final nano-carrier TCPP-MTX-Ag-NP. To prepare the nano-carrier TCPP-MTX-NP without silver modification, AgNO3 solution was not added and, the mixture experienced dialysis after stirring for 8 h.

### Concentrations of MTX and TCPP Measurement

The concentration of TCPP was measured by the ultraviolet-visible (UV) spectrum (UV-2600, Shimadzu, Japan). TCPP (10 μg/mL) in DMF was used as the standard solution. The sample was diluted 100 times by DMF before measurement. The wavelength was 416 nm. The concentration of MTX was measured by the high-performance liquid chromatography (HPLC) method. The C18 column (15 cm, 5 μm, Xbridge, Waters, U.S.A.) was used. The mobile phase was phosphoric acid (0.3%)-acetonitrile (85: 15, v/v) and, the flow rate was 1 mL/min (LC-10AT, Shimadzu, Japan). The wavelength was 302 nm (SPD-10A, Shimadzu, Japan). The sample was diluted 100 times by the mobile phase and, filtered through 0.22 μm. MTX (20 μg/mL) in the mobile phase was used as the standard solution. The injection volume was 20 μL. The concentrations of MTX and TCPP were calculated according to the following equations:

(1) $$\begin{array}{l} \text{Concentration of TCPP (mg/mL)}\\ ={\text{Absorbance}}_{\text{sample}}/{\text{Absorbance}}_{\text{standard}}\\ \;\times 10\times 100/1000 \end{array}$$

(2) $$\begin{array}{l} \text{Concentration of MTX (mg/mL)}={\text{Area}}_{\text{sample}}/{\text{Area}}_{\text{standard}}\\ \;\times 20\times 100/1000 \end{array}$$

The encapsulation efficiency was calculated according to the following equation:

(3) $$\begin{array}{l} \text{Encapsulation efficiency }\left(\%\right)=\text{concentration (mg/mL)}\\ \;\times \text{sample volume (mL)}/4\times 100 \end{array}$$

### The Silver in TCPP-MTX-Ag-NP

The atomic absorption spectrum (AAS) was used to measure silver element in the nano-carrier (ICE3000, Thermo Scientific, U.S.A.). The sample was diluted 50 times by deionized water before analysis.

### Characterizations of the Nano-Carriers

The scanning electron microscope (SEM) was used to characterize the morphology of TCPP-MTX-Ag-NP and TCPP-MTX-NP (JSM-6510, Shimadzu, Japan). Firstly, the samples were freeze-dried to powder, then the powder was gold-coated and imaged by SEM. The voltage was 5 kV. The TCPP-MTX-Ag-NP was further imaged by transmission electron microscope (TEM), the voltage was 100 kV (JEM 2100F, JEOL, Japan). The particle size of TCPP-MTX-Ag-NP and TCPP-MTX-NP were measured by the dynamic light scattering (DLS) method (Nano-ZS, Malvern, U.K.). To study the stability of the nanoparticles, the TCPP-MTX-NP and TCPP-MTX-Ag-NP in saline were stored at 4°C for 3 months. Then, the particle sizes were measured by the DLS method. The X-ray photoelectron spectrum (XPS) was used to validate the silver element in TCPP-MTX-Ag-NP. The Al X-ray source was used in XPS, the tube voltage was 15 kV and the tube current was 12 mA, the diameter beam spot was 500 μm (Escalab 250xi, Thermo Scientific, U.S.A.).

### The Investigation of the Supermolecular Structure

To explain the structure of the supermolecular nano-carriers, the UV spectrum (UV-2600, Shimadzu, Japan) was employed to get the absorbance profiles of the following six samples: MTX (10 μg/mL) in DMF, TCPP (10 μg/mL) in DMF, TCPP-MTX-Ag-NP (MTX: 5 μg/mL) in DMF, TCPP-MTX-Ag-NP (MTX: 5 μg/mL) in deionized water, TCPP-MTX-NP (MTX: 5 μg/mL) in DMF, TCPP-MTX-NP (MTX: 5 μg/mL) in deionized water. The samples were scanned from 350 to 800 nm. Additionally, the nuclear magnetic resonance (1H-NMR) was also used to study the formation mechanisms. The following samples were analyzed: MTX (10 mg/mL) in *d*_6_-DMSO, GA (10 mg/mL) in *d*_6_-DMSO, TCPP (10 mg/mL) in CF_3_COOD, F127 (30 mg/mL) in CDCl_3_, TCPP-MTX-Ag-NP (MTX: 5 mg/mL) in D_2_O and TCPP-MTX-NP (MTX: 5 mg/mL) in D_2_O.

### The Generation of the Reactive Oxygen Species (ROS)

The ROS generation was assessed by the DPBF method. With blank DPBF as control, the free TCPP, TCPP-MTX-Ag-NP and TCPP-MTX-NP were analyzed (n = 6). In details, acetonitrile was used as the solvent. The final concentration of DPBF in the system was 100 μM and, the nano-carriers according to TCPP and Ag were 10 and 1.5 μg/mL, respectively. The samples in 96-well plate were exposed to 620-nm laser (100 mW/cm^2^) for 0, 10, 20, 30, and 40 s. For comparison, the same samples were also subjected to darkness. At each time point, the absorbance at 410 nm was measured to see the absorbance decrease caused by the ROS generation (Epoch, Biotek, U.S.A.).

### The Release of MTX and TCPP

The MTX and TCPP release was measured by the dialysis method. In details, 0.3 mL of TCPP-MTX-Ag-NP was placed in the dialysis bag (cutting molecular weight: 10 kDa), which was immersed in 40 mL of deionized water with SDS (0.2%) (*n* = 4). The experiment was performed at 37°C and, the shaking speed was 100 rpm. At the time points of 1, 2, 3, 4, 6, 8, 10, 12, 14, 16, 18, 21, 24, 30, 48 h, 0.6 mL of the media was withdrawn and the fresh media was added. The concentrations of TCPP and MTX in the dissolution media were analyzed by UV spectrum and HPLC as described above. After filtering through 0.22 μm, 20 μL of the media was directly injected to measure MTX concentration. And, the absorbance of the media at 416 nm was measured to get the TCPP concentration (UV-2600, Shimadzu, Japan).

### The Biocompatibility Experiment

The human liver cell line L-02 was used in this experiment. The cells were cultured in Dulbecco's modified Eagle media (DMEM) at 37°C in the atmosphere with CO_2_ of 5%. The biocompatibility of the nano-carriers was evaluated by live-dead assay and MTT method. In details, 5 × 10^4^ of L-02 cells were seeded in 24-well plate and, cultured overnight for cell attachment. TCPP-MTX-Ag-NP and TCPP-MTX-NP were added to the cells and, the final concentration according to TCPP and Ag were 10 and 1.5 μg/mL, respectively. After incubation for 24 h, the TCPP-MTX-Ag-NP + laser and TCPP-MTX-NP + laser groups were exposed to 620-nm laser for 10 min at 100 mW/cm^2^. Then, the cells were assessed by the live-dead assay using the Calcein/PI kit according to the protocol, with the cells without treatment as control. The excitation and emission wavelength were 450–490/515 nm for Calcein and, 510–550/575 nm for PI (DMi8, Leica, Germany). The biocompatibility of the nano-carriers was further evaluated by the MTT method. 1 × 10^4^ of L-02 cells were seeded in 96-well plate (*n* = 6) and, cultured overnight for cell attachment. Then, TCPP-MTX-Ag-NP and TCPP-MTX-NP were added to the cells to get a serial TCPP concentrations of 0, 2.0, 4.0, 6.0, 8.0, and 10.0 μg/mL. The Ag concentrations in the TCPP-MTX-Ag-NP group was 0, 0.3, 0.6, 0.9, 1.2, 1.5 μg/mL. After incubation for 24 h, the TCPP-MTX-Ag-NP + laser and TCPP-MTX-NP + laser groups were exposed to 620-nm laser for 10 min at 100 mW/cm^2^. Then, the cell viability was measured by MTT method and, the wavelength was 490 nm (Epoch, Biotek, U.S.A.).

### The Anti-bacteria Effect

The use of the bacterial species was approved by Guangzhou Medical University. The Gram-positive *S. aureus* (ATCC 6538) and the Gram-negative *E. coli* (ATCC 35218) were used. The anti-bacteria effect was evaluated as following: 1 mL of the *S. aureus* or *E. coli* (10^6^ CFU/mL) suspension was incubated with TCPP-MTX-NP or TCPP-MTX-Ag-NP (TCPP: 5 μg/mL, Ag: 0.75 μg/mL) for 24 h at room temperature. Then, the bacteria was exposed to 620-nm laser for 10 min at 100 mW/cm^2^. At last, 50 μL of the bacteria was seeded on the Luria-Bertani (LB) plate. The LB plates were placed in an oven of 37°C for 24 h. Then, the LB plates were pictured. At the same time, 50 μL of the bacteria was added to 4 mL of the fresh LB media (n = 4) and, the media was shaking at 37°C with the speed of 250 rpm. The optical density at 600 nm (OD_600_) of the LB media was measured at the time points of 2, 4, 6, 8, 10, and 12 h.

### The Microstructure of *S. aureus* and *E. coli*

One milliliter of the *S. aureus* or *E. coli* (106 CFU/mL) suspension was incubated with TCPP-MTX-Ag-NP (TCPP: 5 μg/mL, Ag: 0.75 μg/mL) for 24 h at room temperature. Then, the bacteria was collected by centrifugation at 300 g for 10 min. The bacteria was fixed by paraformaldehyde (4%) and washed by deionized water. The bacteria was freeze-dried, gold-coated and imaged by SEM. The voltage was 5 kV.

### Statistic Analysis

All values were expressed as mean ± standard deviation (SD). All comparisons were performed by the two-tailed Student's *t*-test. A *p*-value < 0.05 was taken as statistically significant.

## Results and Discussions

### Features of the Supermolecular Nano-Carriers

The supermolecular nano-carriers were simply prepared by mixing the compounds TCPP and MTX in DMF with GA and the polymer F127 in deionized water. The four components will spontaneously construct the nano-structure. The GA served as reducing agent and, reduced AgNO_3_ to provide Ag to the supermolecular nano-carrier. The TCPP-MTX-NP and TCPP-MTX-Ag-NP nano-suspension displayed excellent stability.

**Figure 1:**
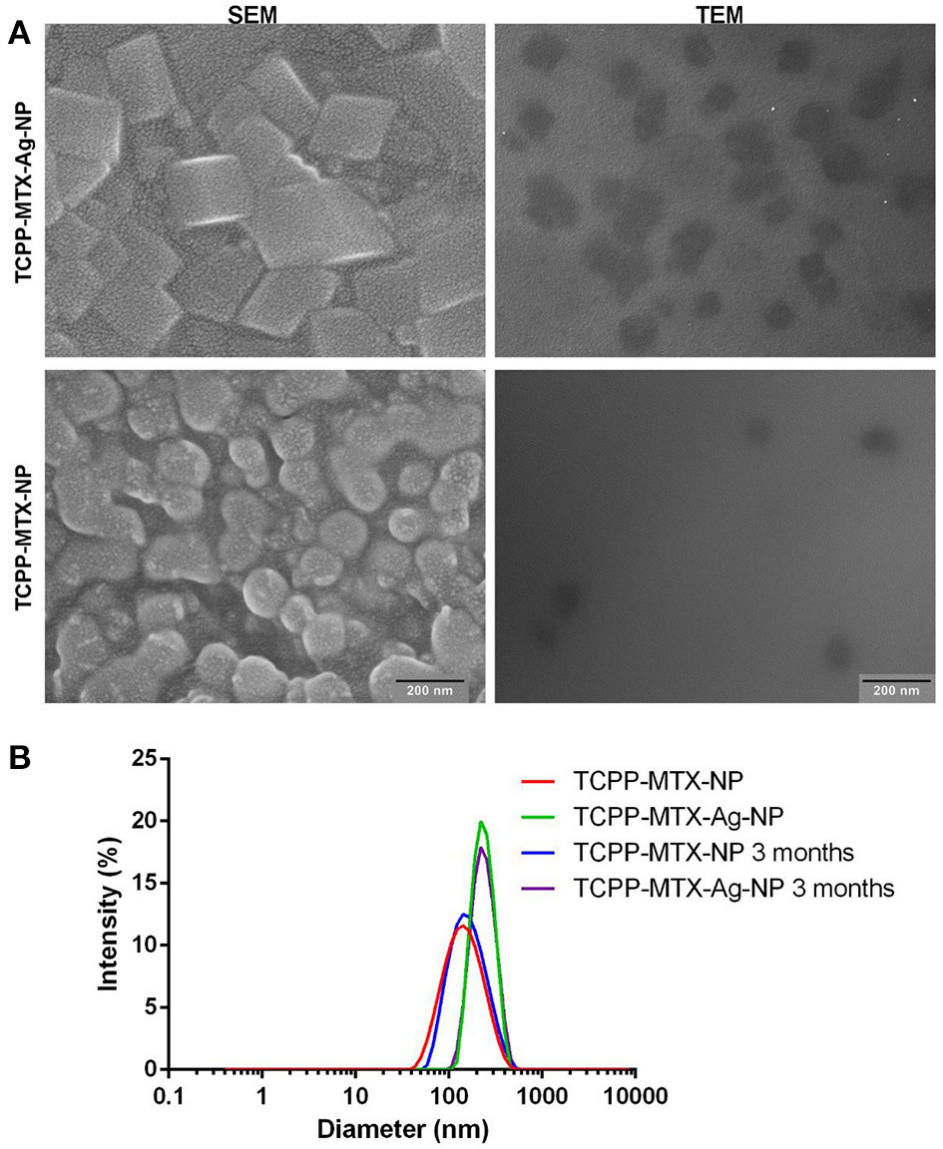
The morphology and size of the supermolecular nano-carriers (the scale bar represents 200 nm). **(A)** The SEM and TEM images of the nanoparticles; **(B)** The size distribution of the nanoparticles in saline before and after storage at 4°C by the DLS method.

The encapsulation efficiency of TCPP and MTX was determined to be ~90 and 85%, respectively. The satisfying encapsulation efficiency indicated that there was a strong driving force for constructing the supermolecular nano-carrier. The silver element analysis by AAS further demonstrated that nearly 100% of the Ag^+^ was successfully encapsulated into the TCPP-MTX-Ag-NP. TCPP-MTX-Ag-NP and TCPP-MTX-NP exhibited distinct morphologies according to SEM observations (Figure 1A). The nano-carrier after Ag-doping was irregular cube-shaped, compared to the spherical shape before Ag-doping. The size of nanoparticles increased after Ag-doping. In a word, the Ag-doping significantly changed the morphology of nano-carrier, which also implied the successful Ag-doping in the TCPP-MTX-Ag-NP. The TEM images further demonstrated the irregular cube-shape of TCPP-MTX-Ag-NP and the spherical appearance of TCPP-MTX-NP and, the size of the former was about 100 and 80 nm for the latter. The size of the nanoparticles shown in SEM images was bigger than that in the TEM images. This phenomenon perhaps was caused by the aggregation of nanoparticles in the freeze-drying process before SEM scanning. The dynamic light scattering (DLS) method was also employed to characterize the nano-carriers. As shown in Figure 1B, the diameter of nano-carriers increased from 156.2 to 237.7 nm after Ag-doping. The increase in size was consistent with SEM and TEM observations. As the DLS method detects the hydrodynamic diameter, the measured size are bigger than those of the SEM and TEM. Furthermore, the self-assembled nano-carriers were stable in saline, the particle sizes of TCPP-MTX-NP and TCPP-MTX-Ag-NP were 167.0 nm and 240.8 nm after storage at 4°C for 3 months.

**Figure 2:**
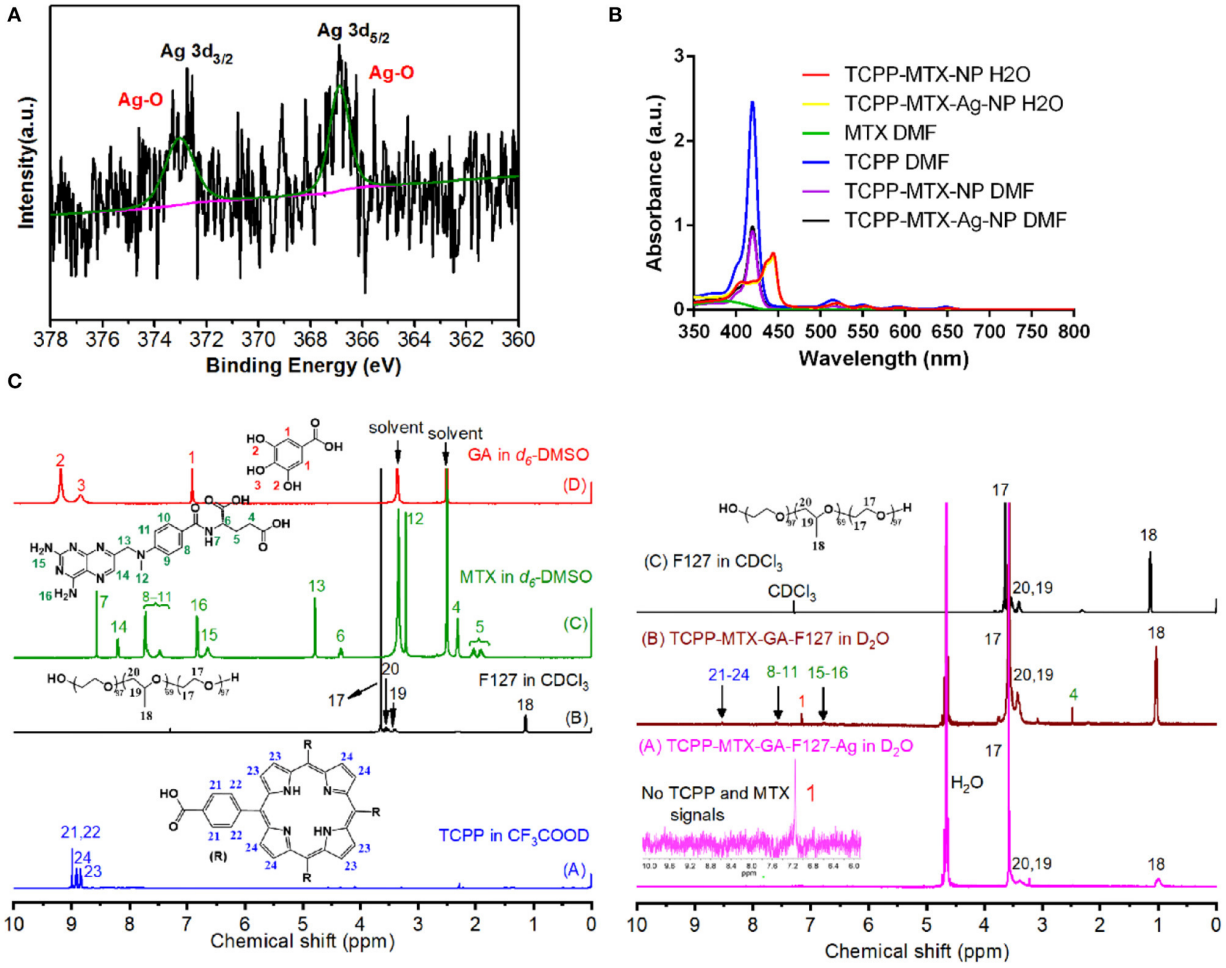
The structure analysis of the TCPP-MTX-Ag-NP. **(A)** The XPS spectrum to show the presence and valance of silver element; **(B,C)** The UV spectrum and 1H-NMR spectrum of different components in the TCPP-MTX-Ag-NP.

The XPS spectrum was used to analyze the silver element in TCPP-MTX-Ag-NP. The XPS spectra of the nano-carrier were collected including the main elements and the expanded patterns of Ag3d. The survey spectrum in Figure 2A clearly showed that Ag was successfully doped into the nano-carrier. The Ag3d spectrum could be deconvoluted into two main peaks at 373.0 and 366.9 eV, originating from Ag-O bond. This may due to the oxidation of sliver element on the surface by oxygen gas.

### Analysis on the Structure of Supermolecular Nano-Carrier

Based on the above results, it was clear that the four components of TCPP, MTX, GA, and F127 spontaneously formed the supermolecular nano-carrier and, the Ag-doping changed the morphology of the nano-carrier. However, the detailed structure of the nano-carriers was still unclear. The UV spectrum and 1H-NMR spectrum were employed to investigate the structure characteristics.

As shown in Figure 2B, there were five peaks at 419, 514, 549, 589, and 647 nm in the spectra of TCPP. With DMF as the solvent, the TCPP-MTX-NP and TCPP-MTX-Ag-NP also displayed the five peaks at the same positions as TCPP. As DMF could completely dissolve the nano-carriers and all the components existed as free molecules in the solvent, the peaks of TCPP did not shift to other positions. In contrast, the TCPP peaks of the nano-suspension of TCPP-MTX-NP and TCPP-MTX-Ag-NP in water shifted to 444, 521, 554, 594, and 652 nm. The red-shift of the peaks implied that a bigger conjugate structure formed in the supermolecular nano-carrier. It also indicated that there were intermolecular interactions between the components and, the interactions forced the formation of the supermolecular nano-carrier.

The structure of the nano-carriers was further analyzed by the 1H-NMR spectrum. As shown in Figure 2C, the free GA molecules in *d*_6_-DMSO displayed the typical peaks of hydrogen element of phenolic hydroxyl group (2, 3) and benzene ring (1). The free MTX molecules in *d*_6_-DMSO displayed the typical peaks of hydrogen element of the amino-group of heterocyclic ring (15, 16) and benzene ring (8–11). The free TCPP molecules in CF_3_COOD exhibited the typical peaks of hydrogen element in the porphyrin ring (23, 24) and benzene ring (21, 22). The free F127 molecules in CDCl_3_ exhibited the peaks of hydrogen element of –CH_3_ (18), -CH_2_ (17, 20) and –CH (19). The TCPP-MTX-NP in D_2_O still displayed all the above peaks of TCPP, MTX, GA and F127. The presence of these peak indicated that all four components existed at the surface of the TCPP-MTX-NP. It was supposed that the phenolic hydroxyl of GA and the oxygen element of F127 formed hydrogen bond, which facilitated the formation of the supermolecular nano-carrier. The TCPP-MTX-Ag-NP in D_2_O only displayed the peaks of F127 polymer and the benzene group of GA. The results indicated that only F127 and GA presented at the surface of TCPP-MTX-Ag-NP and, the Ag-doping buried the MTX and TCPP into the nano-carrier. In other words, the silver layer at the surface of the nano-carrier shielded the signals of MTX and TCPP.

### The ROS Generation of Supermolecular Nano-Carriers

**Figure 3:**
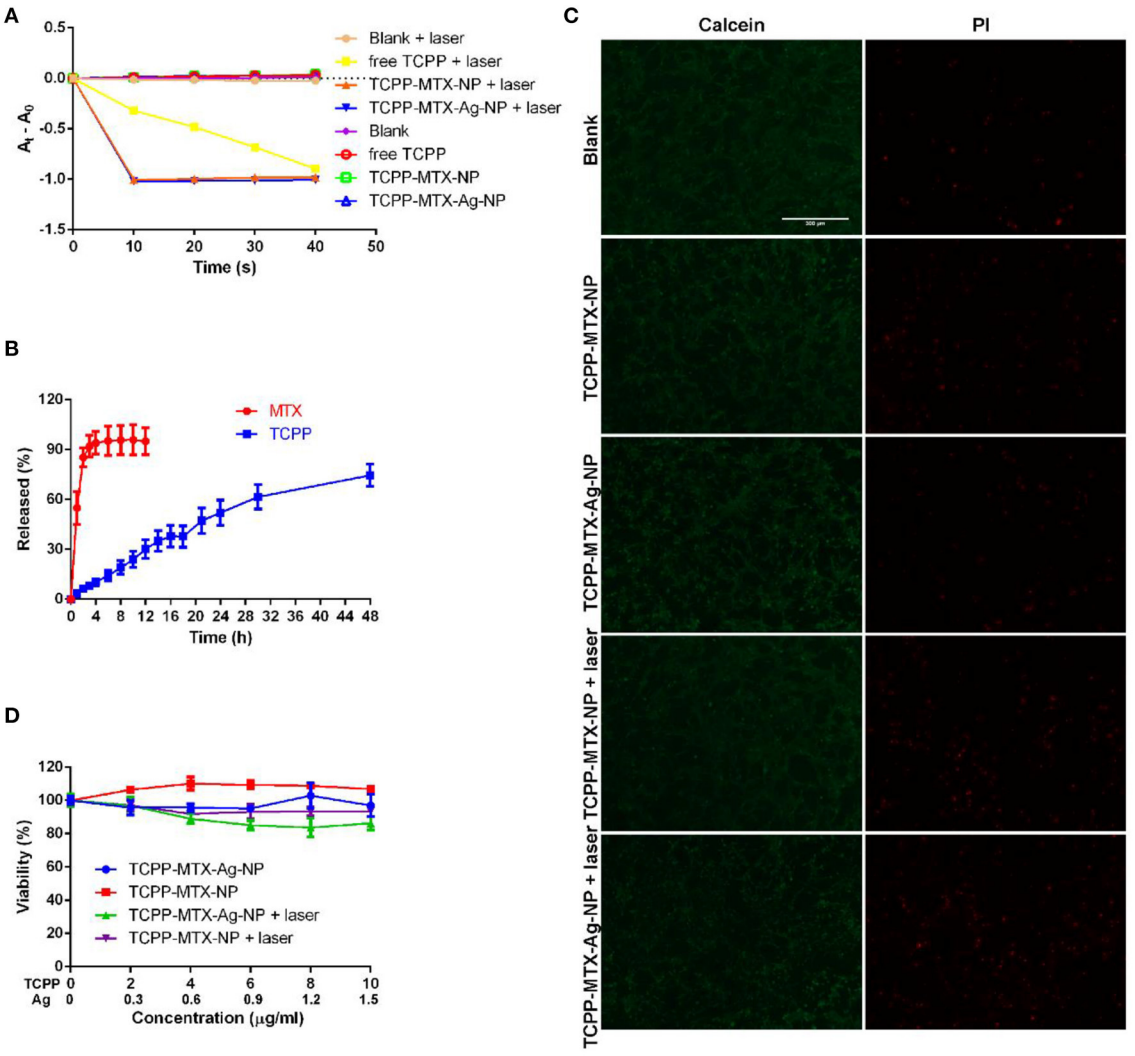
The ROS generation, drug release and biocompatibility of the supermolecular nano-carriers. **(A)** The ROS generation measured by the DPBF method; **(B)** The release of TCPP and MTX from the TCPP-MTX-Ag-NP; **(C)** The biocompatibility assessment by the Calcein/PI experiment (the scale bar represents 300 μm) in L02 cells; **(D)** The biocompatibility evaluation by the MTT method in L02 cells.

The supermolecular nano-carriers were anticipated to play anti-bacteria effects through the direct toxicity of Ag and the ROS generation property of TCPP. The ROS is believed to induce lipid peroxidation and, finally lead to the death of bacteria. In this study, the DPBF was used to assess the ROS generation ability of TCPP-MTX-NP and TCPP-MTX-Ag-NP. The generated ROS will react with DPBF and decrease the typical absorbance of DPBF at 410 nm. As a result, the decrease in absorbance at 410 nm indicates the presence of ROS. As shown in Figure 3A, under the 620-nm laser exposure (100 mW/cm^2^), the DPBF alone exhibited a stable visible absorbance at 410 nm. And, the free TCPP, TCPP-MTX-NP and TCPP-MTX-Ag-NP without laser exposure also displayed a stable visible absorbance. This result indicated that the laser alone or the nanoparticles alone would not produce ROS and validated the applicability of this method. In contrast, with 620-nm laser, the free TCPP induced a steady decrease in absorbance throughout the whole experiment. Both TCPP-MTX-NP and TCPP-MTX-Ag-NP induced a sharp absorbance decrease in the first 10 s and, the total decrease was similar to the free TCPP at the end of the experiment. This experiment clearly validated the ROS generation property of TCPP-MTX-NP and TCPP-MTX-Ag-NP and, it seemed that the nano-carriers could induce more rapid ROS generation than the free TCPP.

### The Release of TCPP and MTX From the Supermolecular Nano-Carriers

To further characterize the TCPP-MTX-Ag-NP, the release of TCPP and MTX from the supermolecular nano-carrier was measured. As shown in Figure 3B, the MTX displayed a rapid release, with 55 and 95% released at 1 and 6 h, respectively. In contrast, the TCPP exhibited a slow release throughout the whole experiment and finally ~75% of the TCPP released at 48 h.

### The Biocompatibility of the Supermolecular Nano-Carriers

Biocompatibility is an important prerequisite of application of the nano-carriers on the body surface. In this study, the normal liver cell line L-02 was used to evaluate the biocompatibility of TCPP-MTX-NP and TCPP-MTX-Ag-NP. The live-dead assay and MTT experiment were employed to evaluate the cellular toxicity. As shown in Figure 3C, at the concentration of 10 μg/mL (calculated based on TCPP), when there was no laser exposure, compared to the blank group, the TCPP-MTX-NP and TCPP-MTX-Ag-NP groups displayed satisfying biocompatibility with the majority of cells alive indicated by Calcein-positive and only a few cells dead indicated by PI-positive. When the cells were exposed to 620-nm laser, the TCPP-MTX-NP and TCPP-MTX-Ag-NP groups still exhibited good biocompatibility. Although the number of the PI-positive dead cells increased, most of the cells were still alive. The excellent biocompatibility was further validated by MTT method (Figure 3D). When there was no laser, both TCPP-MTX-NP and TCPP-MTX-Ag-NP groups displayed good biocompatibility, with nearly all the cells survived after incubation with the nanoparticles for 24 h. When the cells were exposed to 620-nm laser, the viability of the TCPP-MTX-NP group decreased to about 93.5% at the TCPP concentration of 10 μg/mL and, the viability of the TCPP-MTX-Ag-NP group decreased to about 86.2%. This decrease was caused by the photo-toxicity of the TCPP. And, it was supposed that the slight photo-toxicity was acceptable.

### *In-vitro* Antibacterial Activity of Supermolecular Nano-Carriers

**Figure 4:**
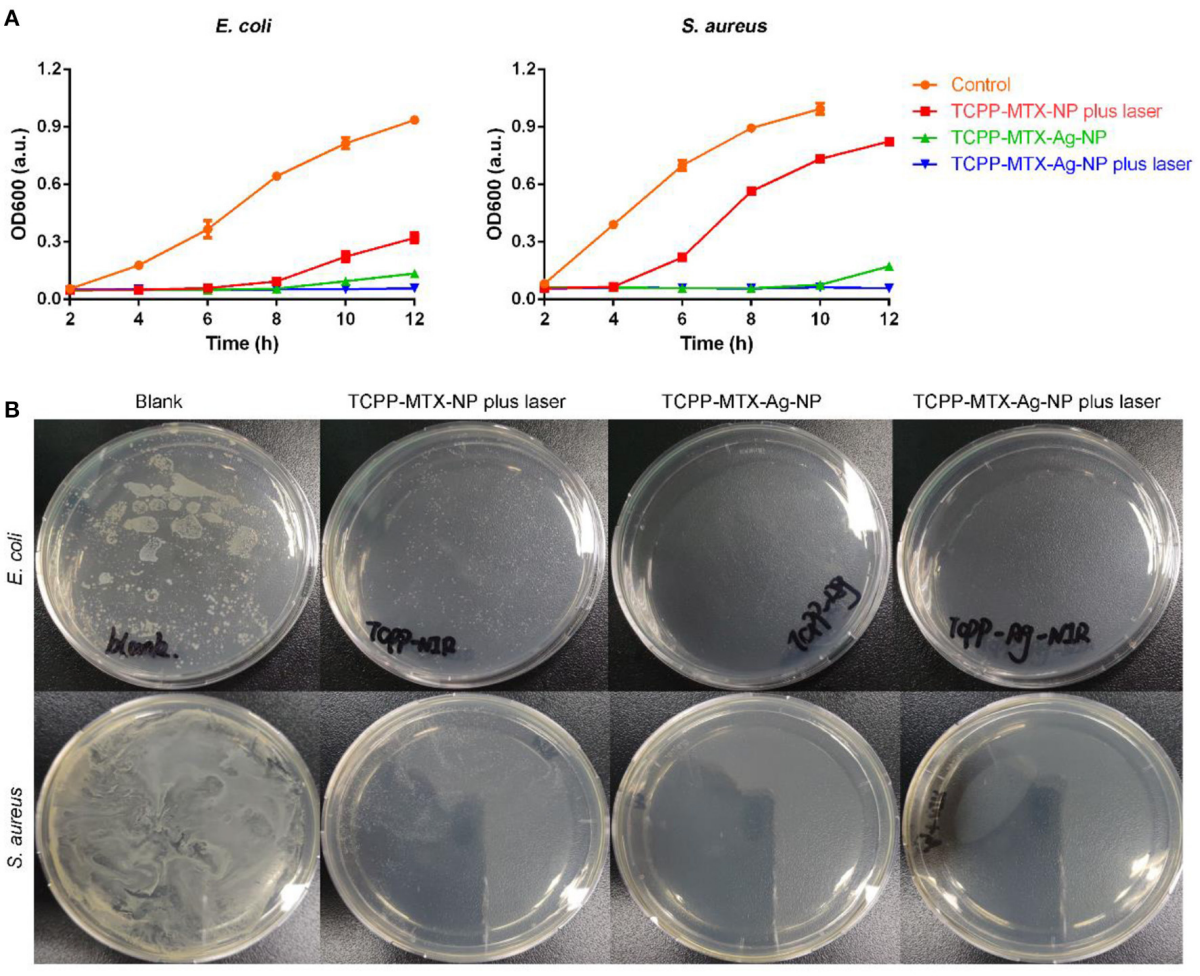
The antibacterial activity of the supermolecular nano-carriers. **(A)** The bacterial growth profiles; **(B)** The bacterial colony formation in the LB plate.

The antibacterial activity of the nano-carriers was evaluated toward the Gram-negative *E. coli* and the Gram-positive *S. aureus*. As shown in Figure 4A, the TCPP-MTX-NP in combination with 620-nm laser significantly inhibited the bacteria growth at the concentration of 5 μg/mL (calculated based on TCPP) and, the inhibitory effect against *E. coli* was more potent than *S. aureus*. The result suggested that the produced ROS of the nano-carrier in response to laser exposure was able to disrupt the bacteria. The TCPP-MTX-Ag-NP displayed stronger antibacterial activity than TCPP-MTX-NP. At the silver concentration of only about 1 μg/mL, the growth of *E. coli* and *S. aureus* was completely inhibited within 8 h even without laser exposure. This indicated that the silver element greatly enhanced the antibacterial effects of the supermolecular nano-carrier. The antibacterial activity of TCPP-MTX-Ag-NP was further improved with the irradiation of 620-nm laser with the growth of bacteria totally blocked throughout the whole experiment. These results demonstrated the synergistic effects of ROS and silver on combating bacterial infection. The LB plates in Figure 4B showed the same trend. Numerous colonies appeared in the control bacteria without any treatment and, the TCPP-MTX-NP plus 620-nm laser treatment obviously reduced the colony numbers. The bacteria colony was completely wiped out by the TCPP-MTX-Ag-NP in combination with laser exposure. In a word, the produced ROS from TCPP-MTX-NP exhibited obvious antibacterial activity and, the silver element in the TCPP-MTX-Ag-NP further enhanced the antibacterial activity.

**Figure 5:**
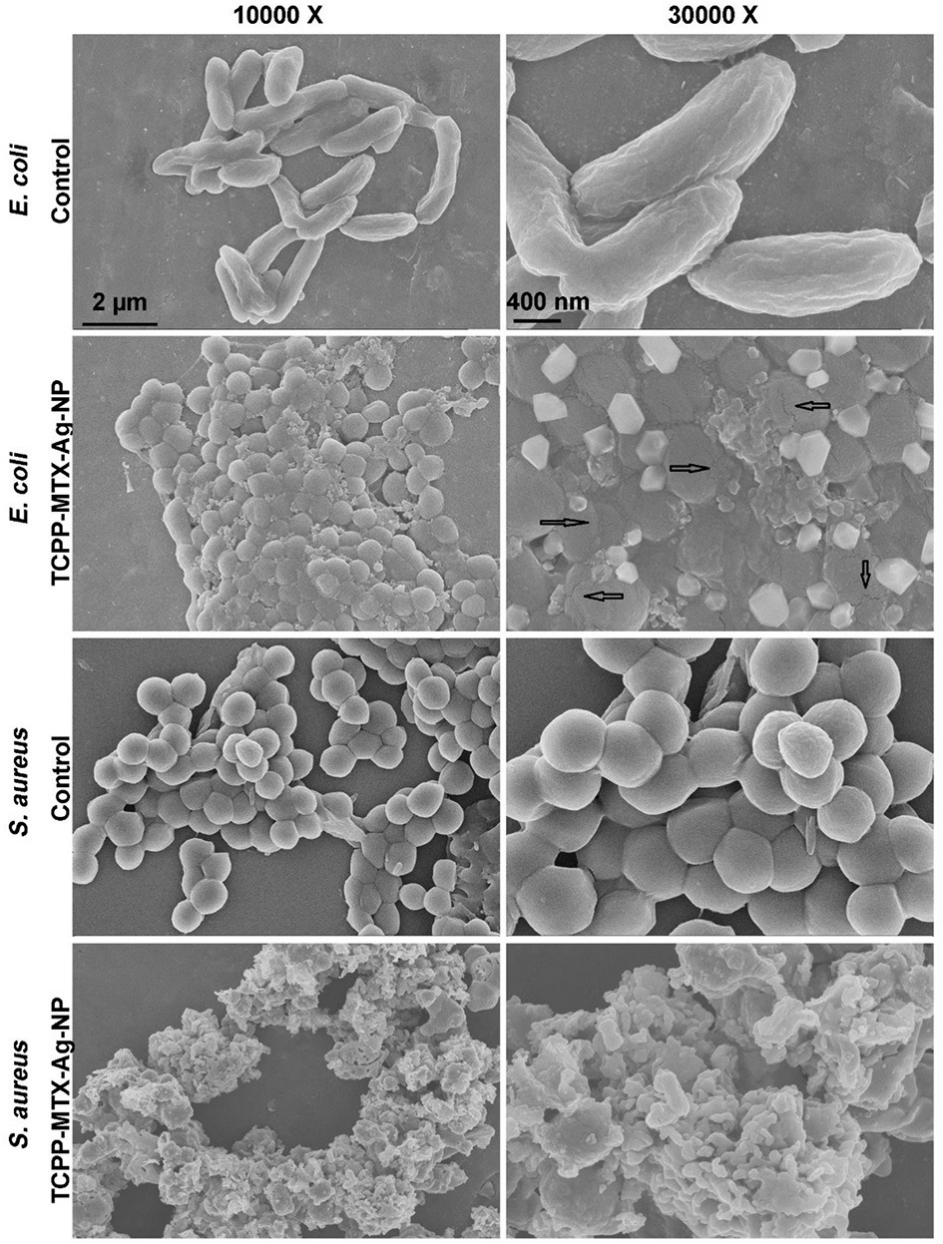
The SEM images of the *E. coli* and *S. aureus*, the arrow indicates membrane rupture (the left scale bar represents 2 μm, the right scale bar represents 400 nm).

The antibacterial potential of TCPP-MTX-Ag-NP was further confirmed by the SEM. As shown in Figure 5, the surface of the control *E. coli* cells was smooth and intact and, the morphology was normal long strip shape. However, after being treated by the TCPP-MTX-Ag-NP, the morphology of the *E. coli* greatly deformed and changed to spherical shape. The phenomenon indicated that the bacteria seriously damaged after the treatment. As indicated by the arrow, the membrane rupture of the bacterial cells can be observed in the magnified SEM image. In addition, the SEM images clearly showed that the plate-like nanoparticles tightly bonded to the surface of the *E. coli*. The control *S. aureus* bacteria showed regular spherical shape and the surface was smooth. After the treatment of TCPP-MTX-Ag-NP, large amount of the nanoparticles bonded to surface of the cells and the surface became wrinkled and rough. In a word, the SEM images demonstrated obvious morphological changes after the TCPP-MTX-Ag-NP treatment, including cell membrane broken, loss of normal appearance, wrinkled and rough surface. Similar phenomena are also observed in other anti-bacterial experiments, caused by silver nanoparticles (Verma et al., 2018) and graphene oxide/cobalt ferrite nanoparticles (Arun et al., 2019). The results clearly indicated the toxic effect of TCPP-MTX-Ag-NP on the integrity of the bacterial cells.

## Conclusions

In this work, the supermolecular nano-carrier TCPP-MTX-Ag-NP was proposed to combat bacterial infection through the toxicity of silver element and the ROS generated from TCPP. Based on the intermolecular interactions among TCPP, MTX, GA and F127, the TCPP-MTX-NP supermolecular nanostructure was successfully constructed. The silver element was further doped into the nanostructure by the reducing effect of GA to form the supermolecular TCPP-MTX-Ag-NP. The nanostructure was validated by the SEM and TEM. The detailed structure of the supermolecular nano-carrier was further analyzed by 1H-NMR, UV spectrum and XPS. The nano-carrier produced ROS under the exposure of 620-nm laser as detected by the DPBF experiment. The TCPP-MTX-Ag-NP displayed excellent biocompatibility with negligible toxicity toward the normal L02 cells. The nano-carrier showed satisfying antibacterial performance through the produced ROS under 620-nm laser and the toxic effects of Ag. The limitation of this study is the lack of investigation on the antiinflammation activity of MTX, which will be studied in the future. In summary, the supermolecular nano-carrier TCPP-MTX-Ag-NP showed excellent antibacterial activity by combining the therapeutic effects of ROS and silver and may serve as a novel strategy of treatment for bacterial infection.

## Data Availability Statement

The original contributions presented in the study are included in the article/supplementary material, further inquiries can be directed to the corresponding author.

## Author Contributions

G-nF, X-tH, and X-lJ carried out most of the experiments. T-wD and Q-xL participated in the DPBF experiment. S-pL corrected the language. J-xL, Q-nW, X-qS, and Y-gH participated in the anti-bacteria experiment. J-jF, LL, and A-pQ proposed the idea and supported the project. All authors contributed to the article and approved the submitted version.

## Conflict of Interest

The authors declare that the research was conducted in the absence of any commercial or financial relationships that could be construed as a potential conflict of interest. The handling editor declared a shared affiliation, though no other collaboration, with one of the authors (T-wD).
